# Without management interventions, endemic wet‐sclerophyll forest is transitioning to rainforest in World Heritage listed K’gari (Fraser Island), Australia

**DOI:** 10.1002/ece3.4853

**Published:** 2019-01-15

**Authors:** Vithya Krishnan, Nicole Robinson, Jennifer Firn, Grahame Applegate, John Herbohn, Susanne Schmidt

**Affiliations:** ^1^ School of Agriculture and Food Science University of Queensland Brisbane Queensland Australia; ^2^ School of Earth, Environmental and Biological Sciences Queensland University of Technology Brisbane Queensland Australia; ^3^ Tropical Forests and People Research Centre University of the Sunshine Coast Maroochydore Queensland Australia

**Keywords:** disturbance, fire, logging, *Lophostemon confertus*, rainforest, *Syncarpia hillii*, tree composition, tree diversity, wet‐sclerophyll forest

## Abstract

Wet‐sclerophyll forests are unique ecosystems that can transition to dry‐sclerophyll forests or to rainforests. Understanding of the dynamics of these forests for conservation is limited. We evaluated the long‐term succession of wet‐sclerophyll forest on World Heritage listed K'gari (Fraser Island)—the world's largest sand island. We recorded the presence and growth of tree species in three 0.4 hectare plots that had been subjected to selective logging, fire, and cyclone disturbance over 65 years, from 1952 to 2017. Irrespective of disturbance regimes, which varied between plots, rainforest trees recruited at much faster rates than the dominant wet‐sclerophyll forest trees, narrowly endemic species *Syncarpia hillii *and more common *Lophostemon confertus*. *Syncarpia hillii *did not recruit at the plot with the least disturbance and recruited only in low numbers at plots with more prominent disturbance regimes in the ≥10 cm at breast height size. *Lophostemon confertus* recruited at all plots but in much lower numbers than rainforest trees. Only five *L. confertus* were detected in the smallest size class (<10 cm diameter) in the 2017 survey. Overall, we find evidence that more pronounced disturbance regimes than those that have occurred over the past 65 years may be required to conserve this wet‐sclerophyll forest, as without intervention, transition to rainforest is a likely trajectory. Fire and other management tools should therefore be explored, in collaboration with Indigenous landowners, to ensure conservation of this wet‐sclerophyll forest.

## 1 INTRODUCTION

Large spatio‐temporal variation, natural and anthropogenic disturbances, and changes in tree population density may lead to different forest communities that are not predicted by current ecological and successional theory (i.e., alternative stable states; Beisner, Haydon, & Cuddington, 2003). For example, the frequency and stochasticity of environmental perturbations determine community resistance and its ability to return to a stable state (Ives & Carpenter, 2007). Many ecosystems depend on regular disturbances for persistence (Attiwill, 1994a), including fire that maintains savannas (e.g., Bond, Woodward, & Midgley, 2004, Russell‐Smith et al., 2013), forest and woodlands of North America (Ryan, Knapp, & Varner, 2013), and tall open forests of southwest Australia (Burrows & McCaw, 2013).

One such fire‐dependent system is wet‐sclerophyll forests that form an ecotone between dry‐sclerophyll forest and rainforest (Peeters & Butler, 2014; Stanton, Parsons, Stanton, & Stott, 2014), and the focus of our study. Australian wet‐sclerophyll forests occur over a large latitudinal range in Australia and are typically dominated by canopy trees in the Eucalypteae tribe and wider Myrtaceae family (Ashton & Attiwill, 1994; Donders, Wagner, & Visscher, 2006) with varying understorey depending on climate and disturbance histories (Donders et al., 2006; Harrington & Sanderson, 1994; King, 1985).

With low fire frequencies, for example, once in 100–350 years, wet‐sclerophyll *Eucalyptus‐*dominated forest in southern Australia favors an understorey of rainforest species and is able to regenerate and maintain canopy tree composition and structure (Ashton & Attiwill, 1994; Attiwill, 1994b; Stanton, Parsons, et al., 2014). A minimum of 100 years between fires is proposed as the required interval to maintain wet‐sclerophyll forests in Tasmania (Gilbert, 1959; Hickey, 1994). Without logging or fire, these forests can transition to rainforest (Hickey, 1994), but whether appropriate fire regimes should be implemented to maintain wet‐sclerophyll forests remains debated. Fire is often negatively perceived by the public and some land managers, and with limited knowledge of forest trajectories, attempts to reintroduce fire regimes for conservation purposes can prove difficult (Stanton, Parsons, et al., 2014; Stanton, Stanton, Stott, & Parsons, 2014). Finding appropriate management regimes is pertinent for numerous reasons including that the loss of wet‐sclerophyll habitats may lead to the decline of endangered fauna such as glider possums (*P. australis* var. *reginae*), powerful owl (*Ninox strenua*), and northern bettong (*Bettongia tropica*; Peeters & Butler, 2014; Stanton, Parsons, et al., 2014).

Motivated by the need to direct conservation efforts, we analyzed long‐term experimental plots of wet‐sclerophyll forest. Such long‐term research sites are rare (Franklin, 1989), but when present, allow examining natural and anthropogenic disturbances over larger temporal scales (Pretzsch, 2010). We follow a successional sequence over 65 years in *Syncarpia hillii–Lophostemon confertus* dominated wet‐sclerophyll forests on K'gari, Australia. This forest (Regional Ecosystem 12.2.4) is an “of concern” regional ecosystem with ~10,000 hectares remaining (Queensland Government, 2017).

A considerable body of literature exists on the Cooloola region (Great Sandy National Park) as the dune chronosequence presents a space‐for‐time substitution of ecosystem development with distinct stages of succession and retrogression (Grimes, 1992; Jones, Sanderman, Allen, Dalal, & Schmidt, 2015; Lawson & Wardell‐Johnson, 2006; Spencer & Baxter, 2006; Thompson, 1992; Walker, Thompson, Fergus, & Tunstall, 1981; Wardle, Bardgett, Walker, Peltzer, & Lagerström, 2008; Yeoh et al., 2017). How natural disturbances or silviculture affect the trajectory of wet‐sclerophyll forests, however, is not well understood.

Prior to European interference from the mid‐19th century, traditional Butchulla landowners managed K'gari's ecosystems. Fires were thought to be frequent (i.e., every two months) and of low intensity, which resulted in a comparatively open understorey (Fensham, 1997). Fire management changed with the takeover of K'gari by timber loggers and led to less frequent and higher intensity fires (Spencer & Baxter, 2006). Today, the larger fires (>100 ha) on K'gari are generally linked to increased fuel load rather than periods of low rainfall (Srivastava et al., 2013). Logging commenced on K'gari in 1863 with the extensive removal of *Araucaria cunninghamii *(hoop pine) and *Agathis robusta* (kauri pine). With the discovery of marine borer resistant *Syncarpia hillii* in the late 1870s, logging intensified with the timber used *inter alia *for the construction of the Suez Canal (Lennon, 2012). Logging continued until December 1991 when the island was recommended for UNESCO World Heritage status (Lennon, 2012).

Here, we used three 0.4‐ha plots that have been studied from 1952 to 2017 to examine how wet‐sclerophyll forest (with *Syncarpia hillii* and *Lophostemon confertus* as main overstorey species) changes in tree species composition after selective logging and intermittent disturbances by fire and cyclone. We hypothesize that the current no/low disturbance regime will lead to (a) the eventual decline of focus species *Syncarpia hillii *and *Lophostemon confertus* as competition by rainforest species increases; and (b) changes in tree species diversity, composition, and relative basal area.

## 2 MATERIALS AND METHODS

### 2.1 Study area

**Figure 1:**
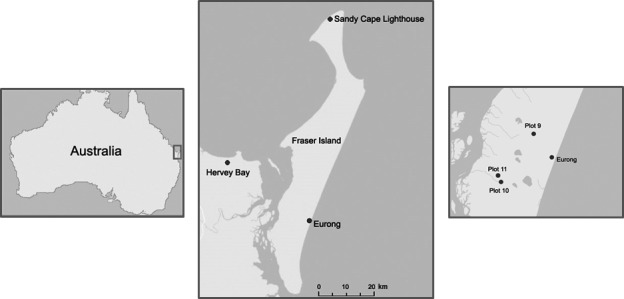
Location of Fraser Island in Australia and study plots

The research was undertaken in *Syncarpia hillii–Lophostemon confertus* forests of K'gari (24°35’–26°20’S and 152°45–153°30’E) located at the southern coast of Queensland, Australia (Figure 1). K'gari is the world's largest sand island, 130 km long and comprising ~168,000 ha (Spencer & Baxter, 2006). The island is characterized by a subtropical humid climate, with mean maximum and minimum temperatures of 25.9°C and 18.8°C, respectively (Sandy Cape lighthouse, Bureau of Meteorology, 2017). Mean annual rainfall ranges from long‐term averages of 1,300 mm at Sandy Cape, to 1,570 mm on the east coast (Eurong), and 1,700 mm on the west coast (Bureau of Meteorology, 2017; Longmore & Heijnis, 1999). Vegetation communities transition from coastal open woodlands on oligotrophic youngest dunes to sclerophyll forest and rainforests on nutrient richer dunes of medium age (Donders et al., 2006; Longmore, 1997). The wet‐sclerophyll forests have myrtaceous canopy species including narrowly endemic *Syncarpia hillii, *more widely distributed *Lophostemon confertus* and *Eucalyptus* species, gymnosperms *Agathis robusta* and *Araucaria cunninghamii*, and N_2_‐fixing *Casuarina torulosa* (Donders et al., 2006). The Queensland Government has listed this regional ecosystem as 12.2.4 and declared it “of concern” for biodiversity status where the *Syncarpia hillii*–*Lophostemon confertus* tall open to closed forest with vine forest understorey (“wet sclerophyll”) occurs on parabolic dunes with an estimated extent of 10,000 ha (Queensland Government, 2017).

### 2.2 Research plots and methodology

We studied forest growth plots (“yield plots”) that were established by the Queensland Forestry Department in 1952 (Joint Conservation Group, 1991). Three plots in *Syncarpia hillii–Lophostemon confertus* (Satinay‐Brushbox) forest were studied with each plot measuring 100 × 40 m (0.44 ha) and mapped with latitudes and longitudes (Table 1). Plots were divided into subplots of 25 × 10 m. Within these subplots, trees with a stem ≥10 cm in diameter at breast height (DBH) were identified to the species level, location within plots mapped, and DBH recorded. The Forestry Department documented plot histories including selective logging, silvicultural treatments, and natural disturbances that varied between plots (Table 1, Joint Conservation Group, 1991). Tree mortality and DBH measurements were made in 1952, 1954, 1958, 1967, 1968, 1975, 1983, and 1989 by the Forestry Department with records of tree recruitment (≥10 cm DBH). The 1989 survey only measured trees with DBH >20 cm, and we excluded this record from the analysis.

**Table 1:** Natural disturbances, silvicultural treatment, and timber harvest in three research plots of *Syncarpia hillii–Lophostemon* *confertus* forests on Fraser Island, Australia. Data are trees ≥10 cm DBH. GPS coordinates were plot 9 (25°28'7.70"S 153° 4'58.00"E), plot 10 (25°33'24.48"S 153°1'24.72"E), and plot 11 (25°32'37.48"S 153° 1'4.29"E)

Plot	Natural disturbances		Silvicultural treatment	Logging year	Stems removed by logging	Volume logged (m^3^/ha)	Basal area in 1952 (m^2^/ha)	Basal area in 2017 (m^2^/ha)	Increase in basal area 1952–2017 (m^2^/ha)
	Fire	Cyclone							
9	Pre‐1935	1975	Large *S. hillii* ringbarked 1931 but most survived	Pre‐1952	8	71	69.0	77.9	8.9
				1975	1	5.9			
				1977	1	2.7			
10	1952	1975	None	Pre‐1952	12	224	45.2	68	22.8
				1966	21	115			
11	Pre‐1935	None	None	Pre‐1952	5	66	36.1	48.8	12.7
				1975	20	83.5			

In late 2016‐early 2017 (2017 in the following), we measured all trees ≥10 cm DBH in the three plots. Trees with DBH <10 cm had not been recorded previously, but were measured by establishing five 5‐m‐radius plots located randomly within each 0.44‐ha plot to track the recruitment of tree species.

### 2.3 History of the three studied plots

Disturbance regimes, logging frequency and intensity, and basal area differed between plots (Table 1). Fire affected all plots pre‐1935, and plot 10 in 1952 reducing undergrowth. Cyclones affected plots 9 and 10 (severely damaged) in 1975 (Joint Conservation Group, 1991). Only plot 9 was subjected to ringbarking with intentions to convert the plot to a hoop pine plantation but most trees survived. All plots were logged (estimated since 1915) prior to commencement of regular monitoring in 1952 with selective removal of old growth trees (Joint Conservation Group, 1991). Plot 10 was the heaviest logged of the three plots pre‐ and post‐1952 (Table 1).

### 2.4 Data analysis

#### 2.4.1 Species diversity

Analyses were performed using the program R (R Core Team, 2017). Species diversity was calculated by using Shannon's index (Magurran, 1988), Pielou's evenness (Pielou, 1975), and species richness for each plot in every year of measurement. The effect of year on Shannon's index and evenness was calculated by using a linear mixed‐effects model with corAR1 correlation structure from the nlme package (Pinheiro, Bates, DebRoy, & Sarkar, 2017), accounting for temporal autocorrelation (Zuur, Ieno, Walker, Saveliev, & Smith, 2009). Relative basal area of trees (≥10 cm) was calculated and graphed on GraphPad Prism 7 (2017).

#### 2.4.2 Changes in species composition

Tree stem maps were generated for each plot as a graphical representation of tree location and DBH sizes (≥10 cm) relative to each plot at the start of the survey period (1952) and at the latest measurement (2017). Non‐metric multidimensional scaling (nMDS) based on the Bray–Curtis similarity index was used to examine the pattern in plant species composition in 1975, 1983, and 2017 among plots with trees ≥10 cm DBH. These three years were chosen to examine the changes between plots after the cyclone in 1975 (Table 1). Data from the 48 subplots (16 (25 × 10 m) subplots in each 0.4‐ha plot) were used for analysis due to a lack of adequate replication.

To visualize the composition of the understorey among plots, nMDS was performed for trees <10 cm DBH in 2017. This was conducted using functions metaMDS() and ordiellipse() in the vegan package (Oksanen et al., 2016) and plotted using ggplot2 (Wickham, 2009). This ordination method is preferred in community ecological studies to determine patterns in multivariate datasets (McCune & Grace, 2002). The following analyses were performed to determine compositional differences between plots. Homogeneity of variance between plots for trees ≥10 cm and <10 cm DBH was assessed using the betadisper() function in vegan, an analogue of Levene's test for homogeneity of variance (Anderson, Ellingsen, & McArdle, 2006). Differences in tree species composition ≥10 cm and <10 cm DBH among plots were determined by using permutational multivariate analysis of variance (PERMANOVA) with the adonis() function in vegan (Anderson, 2001). If adonis() returned significant results, a post hoc multilevel pairwise analysis was performed using the pairwise.adonis() function in the pairwiseAdonis package (Martinez Arbizu, 2017). Species contribution to the Bray–Curtis dissimilarity between plots from 1975 to 2017 was assessed by using simper() in vegan.

## 3 RESULTS

### 3.1 Tree species composition changes in plots from 1952 to 2017

**Figure 2:**
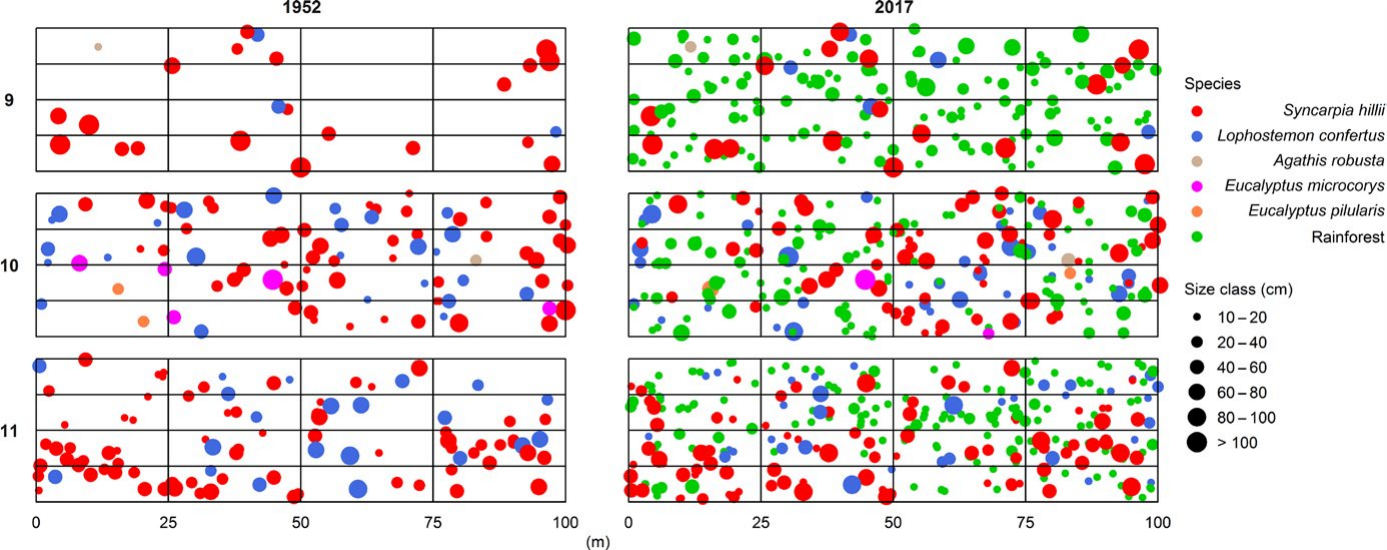
Location of stems ≥10 cm DBH in 1952 and 2017 in 0.4‐ha (40 × 100 m) plot in *Syncarpia hillii–Lophostemon confertus* forests. The five main tree species are indicated by different colors; rainforest species are shown in green (see Figure 2 for details), sizes of dots represent DBH size classes as indicated. Trees <10 cm DBH were not mapped

The trajectory of change in tree composition over 65 years was mapped with tree stems (≥10 cm DBH; Figure 2). The focus species *S. hillii *and *L. confertus* were dominant in all plots in 1952, with an increasing number of rainforest trees recruiting by 2017. In 1952, *S. hillii* was more abundant (20 trees) than *L. confertus *(3 trees) in plot 9 and *S. hillii* trees were on average larger (> 80 cm DBH) than in plots 10 and 11. While plot 10 had smaller individuals of *S. hillii* than plot 9, it had higher abundances of *S. hillii* (60 trees) and *L. confertus* (25 trees). Plot 11 had a similar abundance of focus species as plot 10 with 73 and 22 individuals of *S. hillii *and *L. confertus, *respectively.

By 2017, rainforest tree species, including the abundant *Backhousia myrtifolia *and *Schizomeria ovata*, were the dominant trees in all plots (Figure 2). Rainforest trees represented 148 individuals in plot 9, 118 in plot 10, and 159 in plot 11, mostly in DBH classes 10–40 cm. Plots 10 and 11 had new recruits of the focus species (see *Tree Recruitment* section) and shared a similar abundance of *S. hillii* (70 and 88) and *L. confertus *(40 and 39).

### 3.2 Comparison between plots in 1975, 1983, and 2017

**Figure 3:**
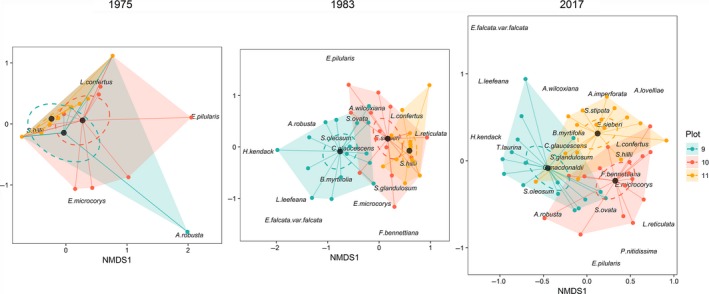
Non‐metric multidimensional scaling (nMDS) of 48 (25 × 10 m) subplots in *Syncarpia hillii–Lophostemon confertus* forests based on abundance of trees ≥10 cm DBH in 1975 (stress: 0.04), 1983 (stress: 0.182), and 2017 (stress: 0.229). The colors represent plots (9: blue, 10: red, 11: yellow). Centroids for each plot are represented by black points. Dashed ellipses represent the 95% confidence interval of centroids. Shaded convex hulls aid in viewing compositional spread

We performed nMDS analyses to visualize compositional changes between plots before the cyclone disturbance in 1975, its resulting effect in 1983, and the current structure in 2017. The nMDS analyses show a divergence in species composition between plots from 1975 to 2017 (Figure 3). The results indicate homogeneity of variance between plots and significant differences in plant composition across years analyzed (Table 2). Post hoc analyses revealed that plot 9 was significantly different in composition from plot 11 in 1975 (*F* value 4.79, *p* = 0.006). Together, *S. hillii* and *L. confertus* explained 96.1% of the total variability in composition between plots 9 and 11 in 1975 (Table 2). SIMPER analyses indicate that plots 10 and 11 were more similar in plant communities (53.4% similar) in 1975 (Table 2).

**Table 2:** Result of analyses performed to determine plant compositional differences between plots in 1975, 1983, and 2017

Analysis	Year					
	1975	1983		2017		
Homogeneity of variance	*F*: 1.02, *p*: 0.368	*F*: 1.82, *p*: 0.174		*F*: 2.96, *p*: 0.06		
PERMANOVA	*F*: 3.25, *p*: 0.008	*F*: 14.2, *p*: 0.001		*F*: 8.87, *p*: 0.001		
Post hoc pairwise adonis	9 versus 10: *F*: 2.97, *p*: 0.108	9 versus 10: *F*: 7.96, *p*: 0.003		9 versus 10: *F*: 10.1, *p*: 0.003		
	9 versus 11: *F*: 4.79, *p*: 0.006	9 versus 11: *F*: 14.9, *p*: 0.003		9 versus 11: *F*: 9.15, *p*: 0.003		
	10 versus 11: *F*: 1.68, *p*: 0.54	10 versus 11: *F*: 2.62, *p*: 0.117		10 versus 11: *F*: 7.11, *p*: 0.003		
SIMPER (influential species driving difference)	9 versus 11:	9 versus 10:	9 versus 11:	9 versus 10:	9 versus 11:	10 versus 11:
	*S. hillii*: 68.5% *L. confertus*: 27.6%	*S. hillii*: 26.3% *B. myrtifolia: *20.4% *S. ovata*: 20% *L. confertus*: 13.7%	*S. hillii*: 33.2% *B. myrtifolia: *19.8% *S. ovata*: 17.3% *L. confertus*: 15.7%	*S. ovata*: 21.2% *S. hillii*: 20% *B. myrtifolia: *17.9% *L. confertus*: 14.1%	*S. hillii*: 24.1% *B. myrtifolia: *19.8% *S. ovata*: 13% *L. confertus*: 11.5% *C. glaucescens: *9.26%	*S. ovata*: 21.3% *B. myrtifolia: *19.7% *S. hillii*: 18.4% *C. glaucescens: *10.7%
	Total: 96.1%	Total: 80.4%	Total: 86%	Total: 73.2%	Total: 77.7%	Total: 70.1%
SIMPER (average dissimilarity between plots)	9 versus 10:60% 9 versus 11:58.5% 10 versus 11:46.6%	9 versus 10:68% 9 versus 11:74.2% 10 versus 11:51.9%		9 versus 10:65.7% 9 versus 11:67.2% 10 versus 11:57.9%		

In 1983, post hoc analyses show that plot 9 differed significantly from plots 10 (*F*: 7.96, *p* = 0.003) and 11 (*F* value 14.9, *p* = 0.003). The species most influential on differences were *S. hillii*, *B. myrtifolia*, *S. ovata*, and *L. confertus* that account for 80.4% difference between plots 9 and 10%, and 86% between plots 9 and 11 (Table 2).

In 2017, all plots had significantly different species composition (Table 2; Figure 3). *S. ovata*, *S. hillii*, *B. myrtifolia*, and *L. confertus* explained 73.2% of the difference between plots 9 and 10. In plots 9 and 11, *S. hillii*, *B. myrtifolia*, *S. ovata*, *L. confertus*, and *C. glaucescens* caused 77.7% of the difference. In plots 10 and 11, *S. ovata*, *B. myrtifolia*, *S. hillii*, and *C. glaucescens* accounted for 70.1% of the difference. Plant community composition differed 65.7% between plots 9 and 10, 67.2% (plots 9 and 11), and 57.9% (plot 10 and 11; Table 2).

### 3.3 Tree recruitment and mortality from 1952 to 2017 (≥10 cm DBH)

Tree mortality varied across plots. Plot 9 had the lowest mortality of the focus species over 65 years (Table 3). One *S. hillii* and one *L. confertus* was removed in plot 9 in logging operations in 1977 and 1975, respectively; the other seven trees presumably died of natural causes. Four out of the seven *S. hillii* trees that had died of presumed natural causes had DBH >100 cm (Table 4).

**Table 3:** Mortality of tree species (≥10 cm DBH) plot from 1954 to 2017 in the *Syncarpia hillii–Lophostemon confertus* forests. Trees either died of natural causes, were logged (denoted by L), or were smashed from logging operations (denoted by S)

Year	*Syncarpia hillii*			*Lophostemon confertus*			*Eucalyptus microcorys*			*Eucalyptus pilularis*			Rainforest species		
	Plot 9	Plot 10	Plot 11	Plot 9	Plot 10	Plot 11	Plot 9	Plot 10	Plot 11	Plot 9	Plot 10	Plot 11	Plot 9	Plot 10	Plot 11
1954						1									
1958			1			1									
1967	2	12 – L 10 – S 3			5 – L 6 – S 1	2		4							
1975	3		12 – L 21 – S	1 – L 1	1	11 – L 11 – S					1				
1977	1 – L														
1983					1	1									
2017	3	3	5		2	4		1					18	4	1

**Table 4:** Diameter at breast height (DBH) size class of *Syncarpia hillii* and *Lophostemon confertus* trees that were logged or died from 1954 to 2017 in each plot

DBH Size class	*Syncarpia hillii*			*Lophostemon confertus*		
	Plot 9	Plot 10	Plot 11	Plot 9	Plot 10	Plot 11
10–20		5 (1967)	17 (1975) 2 (2016)	1 (1975)	5 (1967) 1 (1983) 1 (2017)	1 (1958) 1 (1967) 8 (1975) 1 (1983) 3 (2016)
20–40	1 (1975)	4 (1967) 2 (2017)	3 (1975) 2 (2016)		2 (1967) 1 (2017)	1 (1967) 1 (1975) 1 (2016)
40–60	1 (1975) 1 (1977)	5 (1967) 1 (2017)	1 (1958) 11 (1975)		1 (1975)	1 (1954) 5 (1975)
60–80	1 (1967)	9 (1967)	1 (1975)	1 (1975)	5 (1967)	5 (1975)
80–100		1 (1967)	1 (1975) 1 (2016)			1 (1975)
>100	1 (1967) 1 (1975) 3 (2016)	1 (1967)				2 (1975)

In plot 10, twelve *S. hillii*, five *L. confertus*, and four *E. microcorys *trees were logged and removed in 1966. The logged trees ranged from 50 to 100 cm DBH (*S. hillii*), 60 to 80 cm (*L. confertus*), and 40 to 80 cm (*E. microcorys*). Ten *S. hillii* and six *L. confertus* trees were destroyed during logging in 1966 (with DBH of 10–60 cm, Table 4).

In plot 11, twelve *S. hillii* and eleven *L. confertus *trees were removed by logging in 1975 (Table 3). They ranged from 40 to>100 cm in DBH. Eleven *L. confertus* and 21 *S. hillii* trees were destroyed during logging. The majority of these trees were recruits in the 10–20 cm DBH class (Table 4).

From 1954 to 2017, plot 9 had no recruitment of *S. hillii, *but 36 and 53 *S. hillii* recruited in plots 10 and 11, respectively. The majority of recruitment occurred in the 34 years from 1983 to 2017 (Table 5). Similarly, recruitment of *L. confertus* was low in plot 9 totaling three trees from 1958 to 2017. In plots 10 and 11, 32 and 48 *L. confertus* trees recruited, respectively, between 1954 and 2017, most of which were recorded in 2017 (Table 5). A larger number of rainforest tree species had recruited in plots 9 (65) and 10 (27) in 1983 compared to plot 11 (6). Recruitment of rainforest species had expanded in 2017 to a total number of 95, 94, and 155 trees of ≥10 cm DBH in plots 9, 10, and 11, respectively.

**Table 5:** Recruitment of main tree species (≥10 cm diameter at breast height) in plots that were established in 1952 and monitored over 65 years

Year	*Syncarpia hillii*			*Lophostemon confertus*			*Eucalyptus microcorys*			*Eucalyptus pilularis*			*Agathis robusta*			Rainforest species		
	Plot 9	Plot 10	Plot 11	Plot 9	Plot 10	Plot 11	Plot 9	Plot 10	Plot 11	Plot 9	Plot 10	Plot 11	Plot 9	Plot 10	Plot 11	Plot 9	Plot 10	Plot 11
1954		1	8		1	4												
1958		1	4	1	1	4												1
1967		1	12	1	3	9							1					
1968				1														
1975		3			3	6		1										
1983		5	1		1	5										65	27	6
1989		2			3											6	1	
2017		23	28		20	20		1			1					95	94	155

### 3.4 Species diversity and basal area changes of trees ≥10 cm DBH

**Figure 4:**
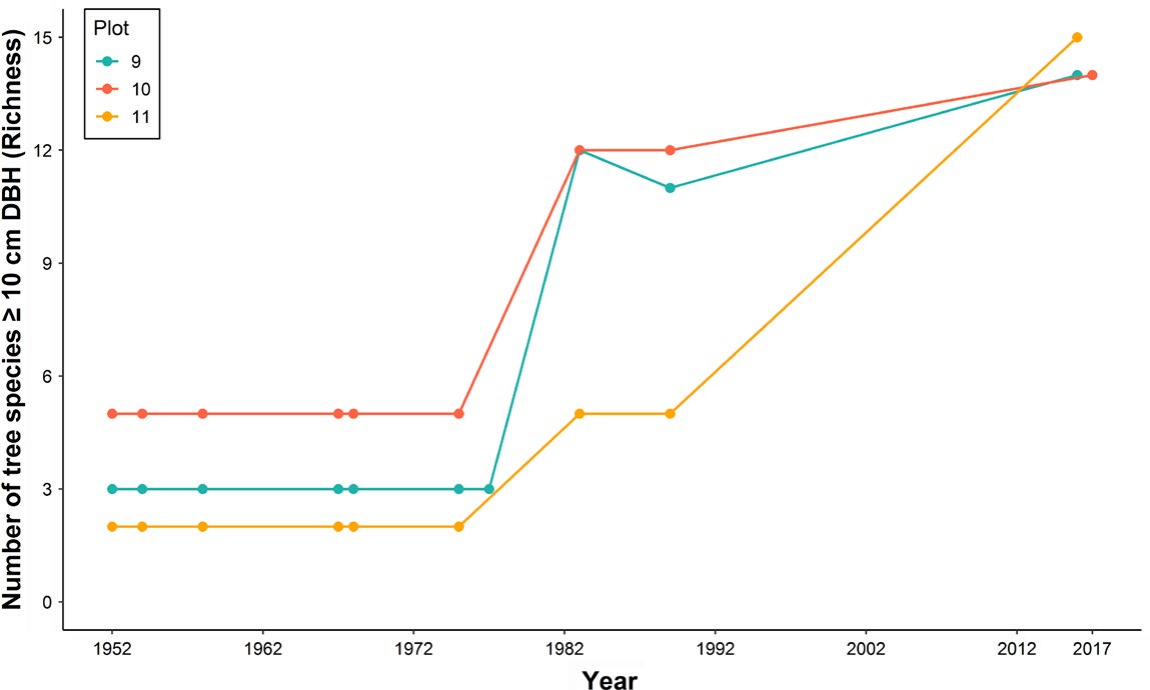
Number of tree species (≥10 cm DBH) recorded from 1952 to 2017 within each plot of the *Syncarpia hillii–Lophostemon confertus* forests. The colors represent plots (9: blue, 10: red, 11: yellow)

Tree species richness (*≥*10 cm DBH) remained relatively low from 1952 to 1975 (Figure 4). Plot 10 contained five tree species during this period; plots 9 and 11 had three and two species, respectively. By 1983, plots 9 and 10 had increased in species richness and totaled 12 tree species, while plot 11 increased to five species. Surveyed in 2017, all plots featured 14 to 15 tree species (DBH *≥*10 cm; Figure 4).

In the 1952 survey, Shannon's diversity indices were 0.51, 0.91, and 0.54 in plots 9, 10, and 11, respectively, and remained relatively stable in all plots until 1975 (Table 6). By 1983, indices had increased to 1.82, 1.51, and 0.84 (plots 9, 10, and 11). By 2017, indices of all plots converged to 1.75–1.99 (Table 6). Significant results from linear mixed‐effects modeling support the increase in Shannon's diversity from 1952 to 2017 after accounting for temporal autocorrelation (estimate = 0.015, *t*‐value = 12.8, *p* = 0).

**Table 6:** Tree diversity (≥10 cm diameter at breast height) based on Shannon's index and evenness in each plot from 1952 to 2017

Year	Shannon's Index			Evenness		
	Plot 9	Plot 10	Plot 11	Plot 9	Plot 10	Plot 11
1952	0.51	0.91	0.54	0.47	0.57	0.78
1954	0.53	0.91	0.55	0.49	0.57	0.79
1958	0.56	0.91	0.56	0.51	0.56	0.81
1967	0.69	0.88	0.58	0.63	0.55	0.84
1968	0.72	0.88	0.58	0.65	0.55	0.84
1975	0.69	0.88	0.54	0.64	0.54	0.78
1977	0.71	NA	NA	0.65	NA	NA
1983	1.82	1.51	0.84	0.73	0.61	0.52
1989	1.79	1.55	0.74	0.75	0.62	0.46
2016–2017	1.99	1.75	1.93	0.76	0.68	0.71

Tree species evenness had similar trends to Shannon's indices over the survey period. In 1952, plots 9 and 10 had an evenness index of 0.47 and 0.57, respectively, and plot 11 of 0.78 (Table 6). In 1983, evenness increased to 0.73 and 0.61 for plots 9 and 10, respectively, while plot 11 decreased to 0.52. By 2017, plots 9, 10, and 11 had similar indices of 0.76, 0.68, and 0.71, respectively. Linear mixed‐effect modeling show a significant increase in evenness from 1952 to 2017 (estimate = 0.005, *t*‐value = 4.55, *p* = 0).

**Figure 5:**
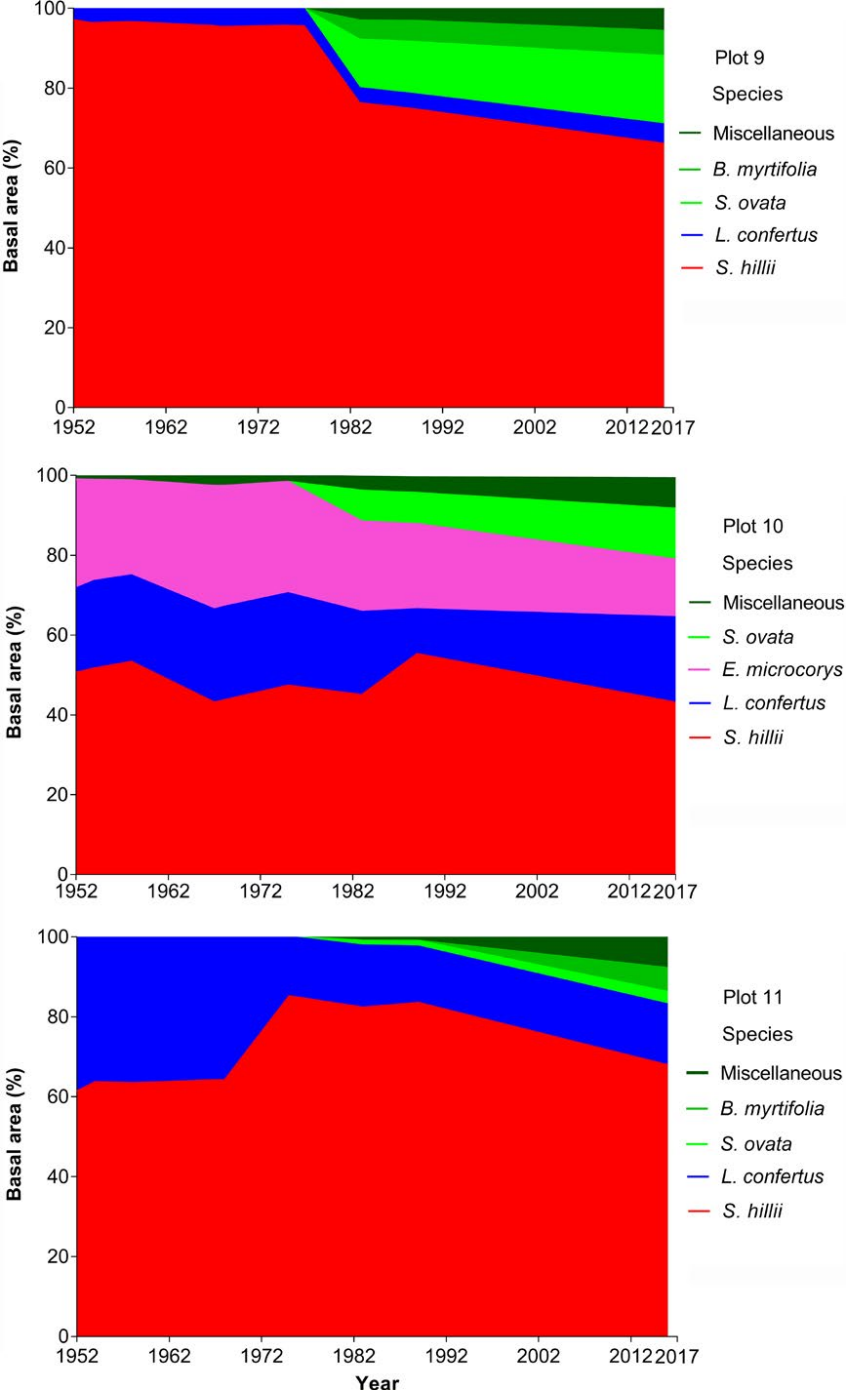
Relative tree basal area (%) from 1952 to 2017 in three plots of *Syncarpia hillii–Lophostemon confertus* forest. Colors represent tree species (red: *S. hillii*, blue: *L. confertus*, light green: *S. ovata*, green: *B. myrtifolia*, dark green: rainforest species)

All plots show different relative basal area (BA) trends with varying degrees of dominance by the focus species. In plot 9, *S. hillii* had been the dominant species over the survey periods with a basal area comprising close to 100% of total BA from 1952 to 1975 (Figure 5). Basal area of *S. hillii* decreased to 76% and 66% of total BA from 1983 to 2017. The BA of *L. confertus* remained low and relatively constant with 3% to 5%. In 1983 and 2017, the rainforest trees *S. ovata *and *B. myrtifolia* had a combined BA of 17% and 23% of total BA, respectively.

In plot 10, the BA of *S. hillii *declined from 50% to 43% of total BA from 1952 to 2017 (Figure 5). The BA of *L. confertus* remained relatively constant throughout the survey period at 21%, while BA of wet‐sclerophyll forest species *E. microcorys *decreased from 27% to 14% from 1952 to 2017. Similar to plot 9, S. *ovata* increased in BA from 7% to 12% of total BA from 1983 to 2017. Other rainforest species increased during this period to account for 6% of overall BA in 2017.

In plot 11, *S. hillii *dominated BA, increasing from 61% to 85% between 1952 and 1975, and declining to 67% in 2017. Basal area of *L. confertus* declined from 38% to 15% of total BA from 1952 to 2017. By 2017, rainforest trees contributed to 16% of overall BA (Figure 5).

**Figure 6:**
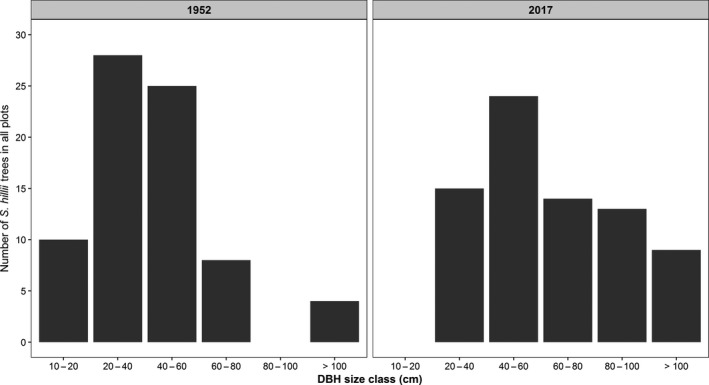
Number of *Syncarpia hillii* trees with full measurements (1952–2017) according to DBH size class (cm) in all plots at the start of the survey period in 1952 and the latest measure in 2017

*Syncarpia hillii* trees that had survived from 1952 to 2017 were analyzed for growth through their size class distribution. In 1952, majority of trees belonged to the 20–40 (28 trees) and 40–60 cm (25 trees) DBH size class, while the 10–20, 60–80, and 100–250 cm size classes had 10, 8, and 4 individuals, respectively (Figure 6). In 2017, the most common size class was 40–60 cm with 24 trees. The 20–40, 60–80, and 80–100 cm size classes had similar tree numbers with 15, 14, and 13, respectively (Figure 6).

### 3.5 Survey and analysis of trees <10 cm DBH

In 2017, trees <10 cm were surveyed in five 78.5 m^2^ subplots within each of the three 0.4‐ha plots to gain a more complete picture of tree recruits. Across the 15 subplots, we measured 260 live trees (<10 cm DBH) with 27 species from 14 families (Table 7). No *S. hillii *recruits were recorded at any plot, and only three and two *L. confertus* recruits were found in plots 10 and 11, respectively. The remaining recruits were species typically associated with rainforests, with all plots containing *Backhousia myrtifolia, Syzygium oleosum, Cryptocarya macdonaldii*, and *Cryptocarya glaucescens.* Between six and 11 species were common across plots.

**Table 7:** Species list and abundance of trees <10 cm diameter at breast height in the three surveyed plots of *Syncarpia hillii–Lophostemon confertus* forest. Gray shading represents low to higher number of individuals

Absent	
1–5	
6–10	
11–20	
>20	

Species	Family	Plot		
		9	10	11
*Backhousia myrtifolia* Hook. & Harv.	Myrtaceae			
*Syzygium oleosum* (F.Muell.) B.Hyland	Myrtaceae			
*Cryptocarya macdonaldii* B.Hyland	Lauraceae			
*Cryptocarya glaucescens* R. Br.	Lauraceae			
*Schizomeria ovata* D.Don	Cunoniaceae			
*Halfordia kendack* (Montrouz.) Guillaumin	Rutaceae			
*Synoum glandulosum var. glandulosum* (Sm.) A.Juss.	Meliaceae			
*Mischocarpus pyriformis *(F.Muell.) Radlk. subsp. *pyriformis*	Sapindaceae			
*Alyxia ruscifolia* subsp. Ruscifolia R.Br.	Apocynaceae			
*Lophostemon confertus *(R.Br.) Peter G.Wilson & J.T.Waterh.	Myrtaceae			
*Endiandra sieberi* Nees	Lauraceae			
*Endiandra discolor *Benth.	Lauraceae			
*Denhamia celastroides *(F.Muell.) Jessup	Celastraceae			
*Acronychia imperforata *F.Muell.	Rutaceae			
*Sarcopteryx stipata* (F.Muell.) Radlk.	Sapindaceae			
*Notelaea longifolia *Vent.	Oleaceae			
*Notelaea longifolia forma glabra *P.S.Green	Oleaceae			
*Polyalthia nitidissima* (Dunal) Benth.	Annonaceae			
*Litsea leefeana* (F. Muell.) Merr.	Lauraceae			
*Litsea reticulata *(Meisn.) F.Muell.	Lauraceae			
*Pittosporum revolutum *Dryand. ex W.T.Aiton	Pittosporaceae			
*Anthocephalus chinensis *(Lamk) A. Rich. Ex Walp	Rubiaceae			
*Trochocarpa laurina* (Rudge) R.Br.	Ericaceae			
*Syzygium johnsonii *(F.Muell.) B.Hyland	Myrtaceae			
*Rhodamnia acuminata *C.T.White	Myrtaceae			
*Gmelina leichhardtii *(F.Muell.) Benth.	Lamiaceae			
*Guioa acutifolia* Radlk.	Sapindaceae			

*Backhousia myrtifolia* recruits were common in plots 9 and 11 with 31 and 63 individuals, respectively, but only five *B. myrtifolia* recruited in plot 10. Nineteen *Syzygium oleosum *recruits were recorded in plot 10, eight in plot 9, and five in plot 11. *Schizomeria ovata* recruits were present in plots 9 and 10 with 11 and eight individuals, respectively, but were absent from plot 11. All three plots contained up to six individuals of *Cryptocarya macdonaldii* and *Cryptocarya glaucescens. Halfordia kendack* and *Synoum glandulosum *were present in plots 9 and 10 with one to eight individuals. *Mischocarpus pyriformis *and *Alyxia ruscifolia* were present in plots 9 and 11 ranging from 1 to 7 individuals. *Endiandra sieberi,* *Denhamia celastroides,* *Acronychia imperforata, Sarcopteryx stipata *and *Notelaea longifolia* were present in plots 10 and 11.

**Figure 7:**
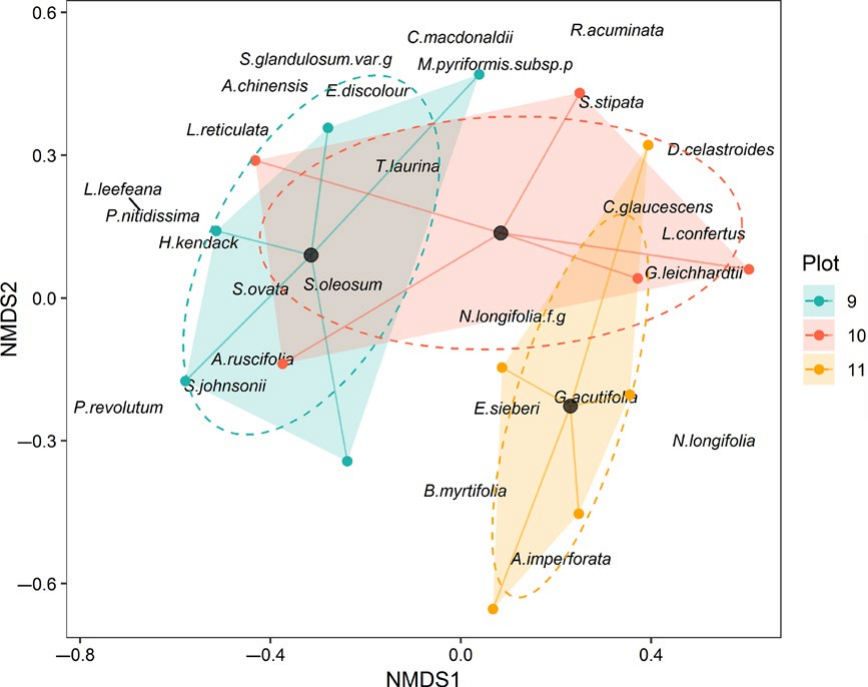
Non‐metric multidimensional scaling (nMDS) of 15 (5 m) radius plots in *Syncarpia hillii–Lophostemon confertus *forests based on abundance of trees <10 cm DBH in 2017 (stress: 0.192). The colors represent plots (9: blue, 10: red, 11: yellow). Centroids for each plot are represented by black points. Dashed ellipses represent the 95% confidence interval of centroids. Shaded convex hulls aid in viewing compositional spread.

PERMANOVA results show significant differences between centroids (*F* value 3.16, *p* = 0.001) indicating differences in species composition between plots in 2017. Dispersion between plots was significantly different (*F* value 5.24, *p* = 0.02) aided by visualization of the nMDS plot (Figure 7). SIMPER analyses show that plots 9 and 10 differed 80.8% in plant species composition, plots 9 and 11 differed 62.2%, and plots 10 and 11 differed 85.5%.

## 4 DISCUSSION

**Figure 8:**
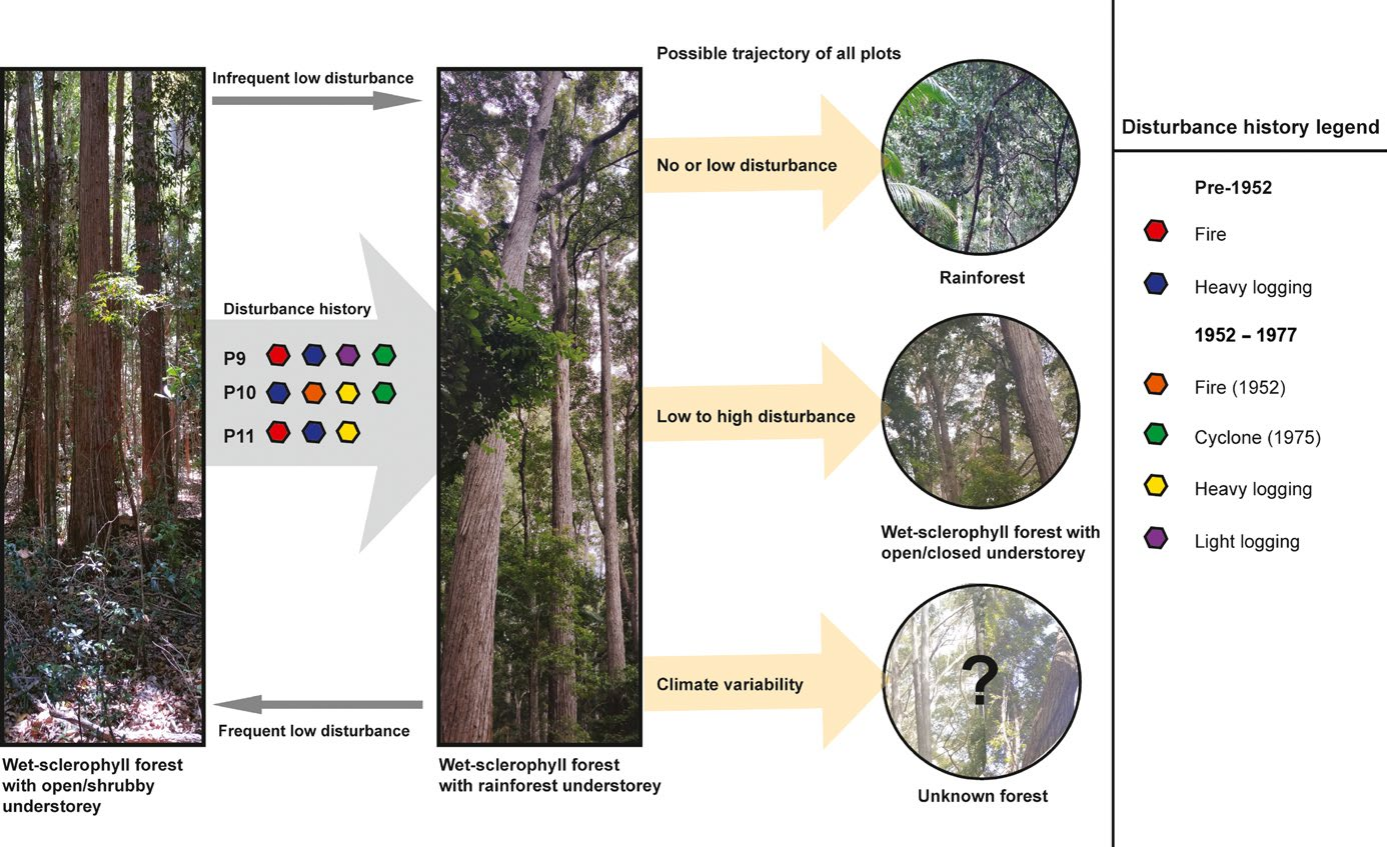
Possible future trajectory of studied plots in the *Syncarpia hillii–Lophostemon confertus forest*

In this study, we had the unique opportunity to track floristic response of wet‐sclerophyll forests of high conservation value over a 65‐year trajectory following different types of disturbance. The studied plots have been subjected to varying degrees of disturbance from before 1952 (from ~1915) to 1975. Long‐term datasets like the one we explore here on forests in Australia are rare and have provided valuable insight into community assembly (Firn, Erskine, & Lamb, 2007; Wills et al., 2018), but their design also presents some limitations as disturbance histories varied and an unlogged forest site was unavailable for comparison. Regardless of disturbance histories, the strong recruitment of rainforest species was accompanied by a concomitant decline of wet‐sclerophyll species from near 100% to between 70% and 82% of tree basal area. The considerable disturbance regimes of logging and fire appear to have facilitated the recruitment of focus species *S. hillii *and *L. confertus*. However, future projections remain unclear as rainforest tree species recruit at considerably faster rates than wet‐sclerophyll species (Figure 8). Throughout Australia, the deliberate use of fire by Indigenous landowners, and subsequent cessation with the displacement of the Indigenous population, has led to widespread change in landscapes and their ecosystems (Gammage, 2012). With the cessation of burning regimes by Butchulla landowners for over a century, and end to silvicultural disturbances since 65 years, it appears likely that this wet‐sclerophyll forest transitions to rainforest. We discuss the implications, limitations, and opportunities resulting from this study.

### 4.1 Mortality and recruitment of focus species *Syncarpia hillii* and *Lophostemon confertus*

Considerable post‐logging mortality of trees (≥10 cm DBH) can occur in tropical and subtropical forests (Nebel, Kvist, Vanclay, & Vidaurre, 2001; Smith & Nichols, 2005). Post‐logging mortality was minor in our plots with two individuals of the focus species and one *E. pilularis* dying as the result of the selective logging within eight years of logging (Joint Conservation Group, 1991). Reasons include that non‐merchantable stems were not poisoned, a common silvicultural practice elsewhere (Smith & Nichols, 2005). Across the studied plots, three large focus trees (>100 cm DBH) died over the 65‐year survey period, 39 years post‐logging, with unknown causes of mortality.

*Syncarpia hillii* and *L. confertus* recruited in the more intensively logged plots 10 and 11 with 91 individuals in 2017 when the survey size of ≥10 cm DBH was reached. Regenerating *E. microcorys* and *L. confertus* in forests in New South Wales (Australia) had a mean annual tree diameter increment of 1 cm (King, 1985). In our study, established *L. confertus *had a mean annual diameter increment of 0.2–0.4 cm. Growth rate depends on resource availability, competition, and facilitation, and is often not linear (Florence, 2004). Recruiting focus species may have commenced their life in the past two decades (assuming 0.4 cm annual increment) or have been seedlings in 1952 (assuming slow initial growth).

The least disturbed of the plots (plot 9), only recruited three individuals of *L. confertus* (≥ 10 cm DBH) and no *S. hillii*. Likely reasons include a lower abundance of smaller individuals of the focus species at the start of the survey (7 individuals between 10 and 40 cm vs. an average of 27 individuals in plots 10 and 11), lower logging intensity (~80 m^3^ timber removed ha^−1^ vs. ~340 and 150 m^3^/ha removed from plots 10 and 11), and no fire for at least 80 years. Only three to five *L. confertus* recruited in the <10 cm DBH class, suggesting that more recent recruitment of the focus species may have been prevented by a lack of seeds and/or unfavorable conditions for seedling establishment and growth.

Collectively, our observations present some evidence that focus species recruitment occurs proportional to disturbance regimes, with selective logging having enabled recruitment of the focus species, although further research is required to assess this notion. If confirmed that the logging regimes rather than fire enables regeneration of wet‐sclerophyll species, it would contrast observations elsewhere where a lack of fire has prevented regeneration of wet‐sclerophyll species (Ashton, 1981; Ashton & Attiwill, 1994; Harrington & Sanderson, 1994; Stanton, Parsons, et al., 2014). Silvicultural regeneration practices in Tasmanian wet‐sclerophyll forests use a combination of clear felling, burning, and sowing of *Eucalyptus *seeds (Baker & Read, 2011; Hickey, 1994). This approach, practised since the 1960s, regenerates *Eucalyptus* species by removing competition from a dense understorey and creating an environment conducive for seedling establishment (Attiwill, 1994b; Baker & Read, 2011; Bassett, Edwards, & Plumpton, 2000). In the mid‐north coast of New South Wales (Australia), wet‐sclerophyll species regenerated irrespective of post‐logging burning, but regeneration was significantly higher after a post‐logging burn (King, 1985). Fire is a requirement for maintaining wet‐sclerophyll forests across broad climate gradients in North Queensland and Tasmania (Hickey, 1994; Stanton, Parsons, et al., 2014), and the lack of post‐logging burning at our study plots may have limited the recruitment of the focus species. High frequency burning every 2–4 years in wet‐sclerophyll forests (dominated by *Eucalyptus pilularis*) in south‐east Queensland increased the mortality of *L. confertus* and *Syncarpia glomulifera*, while a 30‐year absence of fire enhanced their recruitment (Guinto, House, Xu, & Saffigna, 1999). This indicates that regenerative fire intervals ought to exceed decadal scales for wet‐sclerophyll species to reach a size that enables survival.

As outlined above, we observed recruitment of *S. hillii* and *L. confertus* in the more intensively logged plots 65–80 years post‐fire. *L. confertus* is relatively shade tolerant (King, 1985), and *Syncarpia glomulifera *resprouts epicormically after high‐intensity fires (Benson & McDougall, 1998). The focus species in our study are thought to regenerate naturally in a disturbed understorey with increased light availability, but knowledge on germination physiology is lacking (Fitzgerald, 1990). In general, wet‐sclerophyll *Eucalyptus* species are unable to germinate and survive at undisturbed sites, especially where a substantial leaf litter layer covers the soil (Florence, 2004; Florence & Crocker, 1962). Improving knowledge on seed germination, early establishment, and the drivers of tree recruitment across a broader range of disturbance histories will improve predictions of species’ trajectories and the future of *S. hillii–L. confertus* wet‐sclerophyll forest.

### 4.2 Recruitment of rainforest tree species and fire

The fast recruitment of rainforest species relative to the focus species raises concern that rainforest may eventually replace the wet‐sclerophyll forest. While the composition of tree species remained similar prior to 1975, it diverged significantly following the heavy recruitment of rainforest species in more recent decades. This shift in species composition is supported by an increase in Shannon's and evenness indices, with higher diversity attributed to recruitment of rainforest trees. It is possible that recruitment of rainforest trees was triggered by a cyclone disturbance in 1975 that affected plots 9 and 10, with gaps in the canopy allowing rainforest species to establish within eight years. Only minor recruitment of rainforest species occurred in plot 11 during this period. Regardless whether the cyclone played a role in rainforest tree recruitment, the 2017 survey detected the highest rate of rainforest species recruitment (DBH <10 and ≥10 cm) in plots, indicating favorable growth conditions for rainforest trees. The two most common species were pioneer rainforest trees *Schizomeria ovata *and *B. myrtifolia *which occur in the upper strata of rainforests on K'gari (Lennon, 2012). While *S. hillii* remains the dominant species (113 m^2^ BA ha^−1^ in total across plots in 2017), the gradual increase in the basal area of rainforest species from 20 m^2^/ha (1983) to 43.3 m^2^/ha (2017) indicates increased presence (and competition) from rainforest recruits in all plots.

Wet‐sclerophyll forests in north Queensland experience similar encroachment by rainforest species with a narrow wet‐sclerophyll ecotone between *Eucalyptus*‐dominated forest and rainforest (Harrington & Sanderson, 1994; Russell‐Smith & Stanton, 2002; Stanton, Parsons, et al., 2014; Stanton, Stanton, et al., 2014; Unwin, 1989). The required fire intensity to maintain wet‐sclerophyll forests in north Queensland rarely occurs (Ash, 1988) as low to moderate fires extinguish upon reaching rainforest boundaries (Stanton, Parsons, et al., 2014; Unwin, Stocker, & Sanderson, 1985). Rainforest species in north Queensland demonstrate fire resilience similar to most sclerophyllous species, and only frequent fire can inhibit their establishment (Williams, Parsons, Jensen, & Tran, 2012). The absence of fire over 20 years appears to provide an irreversible transition to rainforest at these sites (e.g., Mt Fox and Taravale; Stanton, Parsons, et al., 2014). In southern Queensland, however, high‐intensity fire does not appear to be required for maintenance of wet‐sclerophyll species; instead, irregular low‐intensity fire or mechanical disturbances seem to suffice (Peeters & Butler, 2014). Further research is required to determine whether low to moderate intensity fires provide sufficient disturbance to reduce dense undergrowth and promote the regeneration of wet‐sclerophyll species in our study.

### 4.3 Managing *Syncarpia hillii*–*Lophostemon confertus* wet‐sclerophyll forest

Our study provides some insight into the fate of the two focus species over a 65‐year survey period and indicates that both species are unlikely to sustain their presence over time in an unmanaged forest, that is, in the absence of fire or other interventions. Our findings are in line with the observed decline in overstorey *Eucalyptus* species in unburnt wet‐sclerophyll forests (Ellis, 1985; Harvest, Davidson, & Close, 2008). In Tasmanian wet‐sclerophyll forests, the decline of *Eucalyptus* follows the change in burning regimes of Indigenous landowners from high frequency and low‐intensity fires, to infrequent, high‐intensity fires or fire exclusion. This shift has prompted the expansion of rainforest species, litter accumulation, changes to the soil environment, and decreased presence of *Eucalyptus* species (Harvest et al., 2008). In the same forest, removal of rainforest species (logging and burning) can reverse the deterioration of *Eucalyptus* species (Ellis, Mount, & Mattay, 1980). Fire exclusion confers a competitive advantage to rainforest species as they develop quickly and subsequently reduces the likelihood of fire (Stanton, Parsons, et al., 2014). It appears that the absence of fire promotes faster recruitment of rainforest species than of *S. hillii *and *L. confertus*, transitioning the wet‐sclerophyll forest to a closed‐canopy rainforest. While the pros and cons of rainforest expansion remains debated in Australia (Stanton, Parsons, et al., 2014), the restricted distribution of *S. hillii*–*L. confertus* forests to K'gari and the surrounding Cooloola region makes their conservation a priority. Fire management guidelines by the Queensland Department of Environment and Heritage Protection (2017) states the minimum interval between fires should be 20 years, with the maximum interval unknown for this forest (Regional Ecosystem 12.2.4; Queensland Government, 2017). Fire mapping studies on Fraser Island spanning a 20‐year study period (1989–2008) illustrate that larger fires across the various vegetation communities were associated with increased fuel age, and high fuel loads were predominant in all flammable vegetation types on the island by the end of the study (Srivastava et al., 2013). This amplifies the fire management issue as certain sclerophyllous species may not be able to recover after intense wildfires (Peeters & Butler, 2014). Evaluating the effects of fire regimes, including those of Indigenous owners, should be a priority.

It is more difficult to justify logging to allow recruitment of *S. hillii* and *L. confertus*. Logging intensities and the effect of logging as a surrogate for natural disturbance remain debated (Fitzgerald, 1991). Whether selective logging could be a substitute for fire is unclear, while potential benefits include revenue to assist in managing the forests. Selective logging as a management tool would need to have strong stakeholder agreement of Indigenous landowners, government regulators, and the public, as well as considering K'gari's World Heritage status and forest stewardship certification. Selective logging may be viewed as resurrecting K'gari's past logging regime and a step into the past when the forests were exploited for their timber. Logging has not occurred on K'gari since the cessation of commercial logging 27 years ago. Selective logging is used to manage Tasmania's wet‐sclerophyll forests together with other silvicultural practices (Baker & Read, 2011; Hickey, 1994).

Another factor to consider in the management of these forests is climate variability. Changes in temperature and rainfall may affect tree population densities and competitive abilities of species in the wet‐sclerophyll ecosystem (Peeters & Butler, 2014). Such changes are difficult to predict and their influence on species is not well understood (Peeters & Butler, 2014), but maintaining wet‐sclerophyll forests remains a priority as it provides the maximum carbon and biodiversity benefits at sites suited for this vegetation.

Taken together, our study suggests that the survival of *S. hillii* is dependent on its regenerative capabilities following disturbance. Its endemism to K'gari and adjacent mainland Great Sandy National Park makes this species a conservation priority, and its loss would adversely affect the biodiversity of this ecosystem. K'gari's World Heritage status is based on the unique nature of the island with dynamic soils and vegetation. It is a tourist hotspot which makes it more difficult to implement fire regimes.

Several steps could be taken to better understand the dynamics of the forest ecosystem studied here. Additional plots in the *S. hillii–L. confertus* forests could be studied to evaluate whether the higher recruitment of rainforest than focus species occurs across the island. Long‐term trial plots testing various fire regimes could be implemented to assess the most appropriate fire intensity and interval for management of these forests in collaboration with Indigenous landowners. A thorough assessment is warranted on K'gari and the adjacent mainland region to study this forest type. A broader range of disturbance regimes and environmental conditions should be examined to disentangle the contribution of biotic and abiotic variables on the future of this wet‐sclerophyll forest.

## AUTHOR CONTRIBUTIONS

VK, SS, and JF led the writing of the manuscript, analysis, and interpretation of data. NR, JH, GA, and SS contributed to the design, equipment, acquisition, and analysis of data. All authors contributed to the drafts and gave final approval for publication.

## Data Availability

Raw data containing tree number, tree species and identity, survey year, diameter at breast height (≥ 10 cm and < 10 cm), coordinate within plots, and associated R codes used for analyses have been deposited to Dryad: https://doi.org/10.5061/dryad.p0h2678.
